# Development of a promising antigenic cocktail for the global detection of *Babesia caballi* in horse by ELISA

**DOI:** 10.1371/journal.pone.0284535

**Published:** 2023-04-14

**Authors:** Shimaa Abd El-Salam El-Sayed, Mohamed Abdo Rizk, Hanadi B. Baghdadi, Aaron Edmond Ringo, Gantuya Sambuu, Arifin Budiman Nugraha, Ikuo Igarashi

**Affiliations:** 1 National Research Center for Protozoan Diseases, Obihiro University of Agriculture and Veterinary Medicine, Obihiro, Hokkaido, Japan; 2 Department of Biochemistry and Chemistry of Nutrition, Faculty of Veterinary Medicine, Mansoura University, Mansoura, Egypt; 3 Department of Internal Medicine and Infectious Diseases, Faculty of Veterinary Medicine, Mansoura University, Mansoura, Egypt; 4 Biology Department, College of Science, Imam Abdulrahman Bin Faisal University, Dammam, Saudi Arabia; 5 Basic and Applied Scientific Research Center (BASRC), Imam Abdulrahman Bin Faisal University, Dammam, Saudi Arabia; 6 Zanzibar Livestock Research Institute, Ministry of Agriculture, Irrigation, Natural Resources and Livestock, Zanzibar, Tanzania; 7 Laboratory of Helminthology, Institute of Veterinary Medicine, Mongolian University of Life Science, Ulaanbaatar, Mongolia; 8 Department of Animal Infectious Disease and Veterinary Public Health, Faculty of Veterinary Medicine, Bogor Agricultural University, Bogor, Indonesia; Waseda University: Waseda Daigaku, JAPAN

## Abstract

In this study, we designed novel truncated *Babesia caballi* (*B*. *caballi*) recombinant proteins from the previously used *B*. *caballi* proteins; 134-Kilodalton Protein (rBC134) and Merozoite Rhoptry 48 Protein (rBC48). Then, we evaluated the diagnostic performance of the newly designed proteins when used as a single antigen or when used as cocktail antigen consists of rBC134 full length (rBC134f) + newly designed rBC48 (rBC48t) or newly designed rBC134 (rBC134t) + rBC48t for the detection of *B*. *caballi* infection in horse using indirect enzyme-linked immunosorbent assay (iELISA). We used one dose and a half of each antigen in the cocktail formulas. The serum samples were collected from different endemic areas in addition to the sera collected from horses experimentally infected with *B*. *caballi* were used in the present study. Cocktail antigen in full dose of (rBC134f + rBC48t) exhibited the highest optical density (OD) values with *B*. *caballi*–infected sera and showed the lowest OD values with normal equine sera or *B*. *caballi*, and *Theileria equi* mixed infected sera in comparison with the single antigen. Interestingly, the same cocktail antigen exhibited the highest concordance rate (76.74%) and kappa value (0.79) in the screening of 200 field serum samples collected from five *B*. *caballi* endemic countries, including South Africa (n = 40), Ghana (n = 40), Mongolia (n = 40), Thailand (n = 40), and China (n = 40) using iELISA and the results were compared to those of indirect fluorescent antibody test (IFAT) as a reference. Moreover, the identified promising cocktail full dose antigen (rBC134f + rBC48t) showed that it can detect the infection as early as the 4^th^ day post-infection in sera collected from experimentally infected horses. The obtained results revealed the reliability of the rBC134f + rBC48t cocktail antigen when used in full dose for the detection of specific antibodies to *B*. *caballi* in horses which will be useful for epidemiological surveys and control of equine babesiosis.

## Introduction

Equine piroplasmosis (EP) is a tick-borne disease of equines caused by the eukaryotic hemo-parasites mostly *Theileria equi (T*.*equi)*, and *Babesia caballi* (*B*. *caballi*). The disease has impact on economy and animal welfare related to limitations in horse transport between endemic and non-endemic regions, reduced performance in sport horses and treatment costs [1]. EP is endemic in most parts of the world, with 33.17% and 20.45% global prevalence of *T*. *equi* and *B*. *caballi*, respectively [2].

*Babesia caballi* is a tick-borne protozoan parasite that infect horses and causes anemia, hemoglobinuria, jaundice, fever, lethargy, abdominal inflammation, and weakening during an acute infection [3]. Moreover, in chronic infection, the horses exhibited limited exercise tolerance, splenomegaly, weight loss, and transient fever [4]. However, due to nonspecific symptoms of *B*. *caballi* infection in horses, the diagnosis based on clinical indicators is difficult. Although, microscopy can be used to make a clear diagnosis during the acute phase of the disease, it is difficult to do so in recovered animals who still carries the parasite [5, 6]. Since *B*. *caballi*-infected animals are susceptible to *T*. *equi* infection, serological differentiation of the two illnesses is critical for prophylactic therapy, epidemiological surveillance, and babesiosis control. The sero-diagnoses by immunofluorescent antibody test and iELISA are suitable for detecting antibodies in subclinical cases or chronically infected animals with markedly low parasitemia [7–9]. However, until now, no single recombinant ELISA containing a single antigen has managed to detect all IgG- or immunoglobulin M-positive samples examined across different stages of the disease. There are different theories for this failure which include, first, the humoral immune response varies with the stage of infection [3]. Second, the used expression vector and protein purification methods affect a recombinant protein’s capacity to reconstitute native epitopes when synthesized in *E*. *coli* [3, 10]. Hence, some antibodies present at one stage of infection may be absent in the other stages and vice versa [8]. This requires multiple epitopes from different antigens to be available in an immunoassay to detect the antibodies present at different disease stages. In this regard, cocktail-ELISA is an effective developed diagnostic tool that possesses high diagnostic sensitivity [3, 7]. Contrary to single antigens, the cocktail formula contains multiple epitopes from different antigens that can diagnose infection in different stages. Therefore, combining two or more recombinant proteins will increase the sensitivity of recombinant-ELISAs, as has been previously determined for toxoplasmosis [10] and schistosomiasis [11], *T*. *equi* [7] and *Babesia bovis* [3] infections resulting in improving specificities, positive predictive values and kappa values as compared with using a single antigen [3, 10]. Although this technique is highly sensitive in diagnosing infection, it has never been used for the diagnosis of *B*. *caballi* infection. Many previous works have identified Bc134-Kilodalton Protein or Bc48 as an antigen recognized by most serums of *B*. *caballi-*infected horses. This indicated that the host has strong humoral immunity against these proteins. Bc48 is one rhoptry protein of merozoites of *B*. *caballi*, which was previously evaluated as a promising antigen for the serological detection of antibodies of *B*. *caballi* [12]. Moreover, BC134 protein was developed and proved to be highly specific for the detection of *B*. *caballi*-specific equine antibodies [13].

In the present study, we designed novel truncated *B*. *caballi* recombinant proteins from the previously used *B*. *caballi* proteins; 134-Kilodalton Protein (rBC134), and Merozoite Rhoptry 48 Protein (rBC48) and evaluated their diagnostic efficacy for global detection of *Babesia caballi* in horses by ELISA when used in cocktail formula. The purpose was achieved using a panel of the *B*. *caballi* experimentally infected sera that had been collected serially from one horse during the course of infection, and field sera samples collected from *B*. *caballi* infected horses in endemic areas [1] (South Africa, Ghana, Mongolia, Thailand, and China) were used.

## Materials and methods

### Institutional review board statement

The Guiding Principles for the Care and Use of Research Animals Declared by Obihiro University of Agriculture and Veterinary Medicine was applied to all animal experiments used in this study. The protocol was approved by the Committee on the Ethics of Animal Experiments of Obihiro University of Agriculture and Veterinary Medicine (Permit number 23–26). The pathogen experiment’s ID was 201910–2.

### Parasites

*Babesia caballi* (U.S. Department of Agriculture [USDA] strain) was grown in horse erythrocytes in continuous microaerophilic stationary-phase cultures using RPMI 1640 medium (Sigma-Aldrich, Japan) [14, 15].

### Sera samples

A panel of the *B*. *caballi* experimentally infected sera that had been collected serially from one horse during the course of infection at 0, 4, 6, 12, 18, 24 and 30 days post infection [7, 16]. In determination of the diagnostic efficacies of the used antigens for the differentiation of *B*. *caballi*, and *T*. *equi* infections, 6, and 4- sera samples collected from *B*. *caballi*-, and *T*. *equi*- experimentally infected horses were used. In addition, field samples collected from equine piroplasmosis-endemic areas (n = 200), including South Africa (n = 40), Ghana (n = 40), Mongolia (n = 40), Thailand (n = 40), and China (n = 40) were used for the calculation of the specificity and sensitivity of indirect enzyme-linked immunosorbent assay (iELISA). Moreover, it was used for the comparison of iELISA and indirect fluorescent antibody test (IFAT) results. Samples were randomly collected in horses under sterile condition and kept in vacutainer tubes without anticoagulants. No vaccination histories were available for these animals, as these animals were derived from rural communities. The blood samples were collected from apparently clinically healthy horses.

### Recombinant bacterial proteins production

Full-length BC134f in addition to truncated BC134t and BC48t genes were amplified using six oligonucleotide primers (Table 1) from a *B*. *caballi* cDNA phage expression library by standard PCRs. Thereafter, suitable restriction enzymes EcoR I and Xho I were used for digestion of the amplified DNA, and then ligated into the EcoR I and Xho I sites of a pGEX-4T *Escherichia coli* (*E*. *coli*) expression plasmid vector (Amersham Pharmacia Biotech, Little Chalfont, Buckinghamshire, United Kingdom). The resulting plasmids, designated pGEX/BC134f, pGEX/BC134t, and pGEX/BC48t, were transformed into the *E*. *coli* BL-21 strain for the production of recombinant proteins.

**Table 1:** Gene-specific primers for amplifying Bc134, Bc134t, and Bc48t.

Gene oligonucleotide primer	Amino acids size	Gene oligonucleotide primer	References
**Bc134f**	(144–3137)	5`- CAGGATCCGTCATGATGAGA GGGAAC -3` 5`- GCGAATTCGGCCTTAGTTGCTGGGG -3`	Tamaki et al. [17]
**Bc134t**	(275–562)	5`- GCGGATCCGTAGCCAAGGCAAAGCCT -3` 5`- GCCCTCGAGCGGCCTTCAAGTTGTTCC -3`	This study
**Bc48t**	(270–458)	5`- GCGGATCCTATAAGAAGTGGTACATGAAGCTG -3` 5`- GCCCTCGAGCTATTTCTCCAATAAATTATCGGC -3`	This study

### Sodium dodecyl sulfate-polyacrylamide gel electrophoresis (SDS-PAGE) and Western blot analysis

SDS-PAGE and subsequent Coomassie Blue staining R250 were used to confirm the generated recombinant protein, and Western blot analysis was used to establish the protein’s antigenicity as previously described [3, 17].

### iELISA

rBC134 full length and rBC134 and rBC48 truncated proteins were used separately and in a cocktail formula; The full dose of (3 μg/ml rBC134f+ 3 μg/ml rBC48t) and (3 μg/ml rBC134t+3 μg/ml rBC48t) together with the half dose of (1.5 μg/ml rBC134f+ 1.5 μg/ml rBC48t) and (1.5 μg/ml rBC134t + 1.5 μg/ml rBC48t) were used for the detection of infection in a panel of the *B*. *caballi* experimentally infected sera that had been collected serially from one horse during the course of infection in addition to field samples collected from *B*. *caballi* infection-endemic areas. The 96-well microtiter plates (Nunc, Roskilde, Denmark) were coated overnight at 4°C with 50 μL of each recombinant protein per well in a coating buffer (50 mmol/L carbonate-bicarbonate buffer, pH 9.6). The plates were washed once with 0.05% PBS-T and then incubated with 100 mL of a blocking solution (3% skim milk in PBS) for 1 h at 37°C. The plates were incubated with 50 μL of the serum samples diluted 1:100 with the blocking solution for 1 h at 37°C. Following six PBS-T washes, the plates were incubated with a secondary antibody 50 μL of goat anti-horse IgG antibody (Bethyl) diluted 1:4000 with the blocking solution for 1 hour at 37°C as secondary antibody. The plates were washed six times as described above and 100 μL of a substrate solution [0.1 mol/L citric acid, 0.2 mol/L sodium phosphate, 0.3 mg/mL of 2, 2- azide-bis (3-ethylbenzthiazoline-6-sulfonic acid) (Sigma) and 0.01% of 30% H_2_O_2_] was then added to each well. Following incubation for 1 h at room temperature, the optical density (OD) was measured with an MTP-500 microplate reader (Corona Electric, Tokyo, Japan) at a wavelength of 415 nm. The experiment was conducted in two replicates. Each sample’s ELISA result was calculated by deducting the mean OD value of the two readings with GST protein from the mean OD value of two readings with the rBC-134f, rBC-134t, rBC-48t proteins and a cocktail of (rBC134f+ rBC48t) and (rBC134t+rBC48t) full and a half doses, using receiver operating characteristic curve analysis with MedCalc statistical software (version 11.4), cutoff values were calculated based on the used noninfected equine sera [18].

### IFAT

The *B*. *caballi–*infected RBCs were coated on IFAT slides (Matsunami Glass Ind., Ltd., Osaka, Japan). Then, the slides were dried and fixed in absolute acetone for 20 min for standard IFAT observation [6]. Briefly, 200 field serum samples diluted in PBS (1:100) were applied as the first antibody on the fixed smears and then incubated for 1 h at 37°C in a moist chamber. After that, the slides were washed with PBS three times, and a secondary antibody, fluorescein isothiocyanate (FITC)–conjugated goat anti-equine IgG antibody (Bethyl Laboratories, Montgomery, TX, USA), was applied at a dilution of (1:250) as recommended by the manufacturer and incubated for 1 h at 37°C. Next, the glass slides were washed two times with PBS and then mounted with 10 μl of a 50% (vol/vol) glycerol–PBS solution. Finally, the mounted glass slides were examined using a fluorescence microscope (E400 Eclipse; Nikon, Kawasaki, Japan). Positive and negative control serum samples for *B*. *caballi* obtained from our laboratory were used for IFAT experiment.

### Statistical analysis

The percentages of agreement, sensitivity, specificity, and kappa values with a 95% confidence interval of the iELISA results were calculated as previously described by [3, 7, 11]. The following kappa values were used to assess the degree of agreement between the iELISA and the IFAT: fair (0.21–0.40), moderate (0.41–0.60), and significant (0.61–0.80). (http://faculty.vassar.edu/lowry/VassarStats.html).

## Results

### Production of *Babesia caballi* recombinant proteins

rBC134f, rBC134t, and rBC48t proteins were successfully expressed in *E*. *coli* with molecular masses of 160, 31.5 and 46 kDa, respectively (S1 Fig; S1 Raw images). The high antigenicity of both proteins was exhibited in Western blot analysis when sera collected from the horse experimentally infected with *B*. *caballi* specifically reacted to the recombinant proteins but not to the control GST protein (S2 Fig; S1 Raw images).

### Application of a *Babesia caballi* cocktail antigens for serological diagnosis

**Fig 1:**
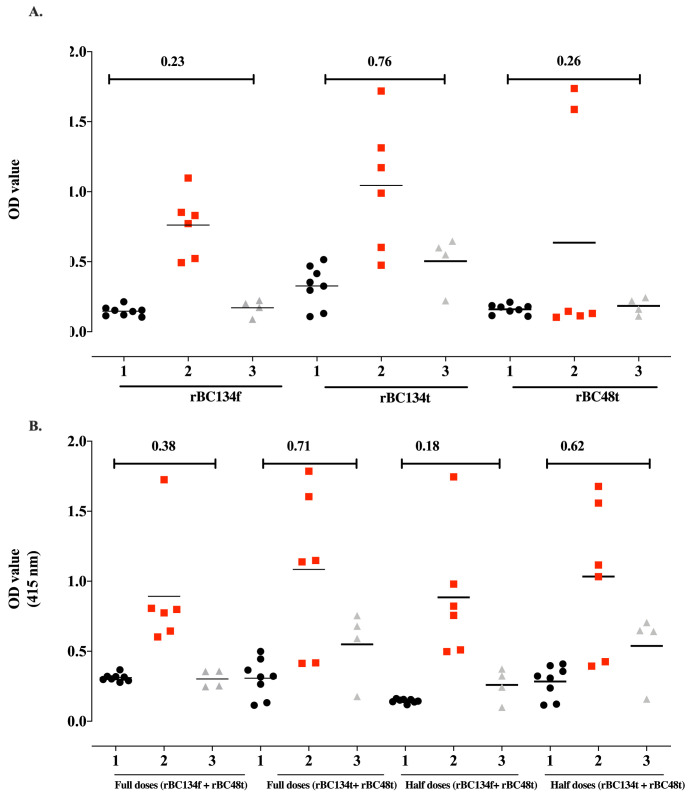
Reactivity of the iELISA using recombinant proteins with horse sera. Lanes: 1, uninfected horse sera (black circle); 2, sera experimentally infected with *B*. *caballi* (red square); 3, sera experimentally infected with *T*. *equi* (Grey tri-angle). The cutoff of each recombinant protein is indicated by a bar. The serum samples were collected at day 18 post infection.

The cutoff OD values for rBC134f, rBC134t, and rBC48t proteins were determined to be 0.23, 0.76, and 0.26 respectively. While for the cocktail formula of full dose (rBC134f + rBC48t), full dose (rBC134t + rBC48t), half dose (rBC134f + rBC48t) and half dose (rBC134t + rBC48t), the cutoff OD values were 0.38, 0.71, 0.18, and 0.62, respectively (Fig 1).

All antigens used either in single or in cocktail formulas clearly differentiated between *B*. *caballi–*infected sera and both *T*. *equi-* and the negative control sera and full dose (rBC134f+ rBC48t) antigen exhibited the highest differentiation (Fig 1).

**Fig 2:**
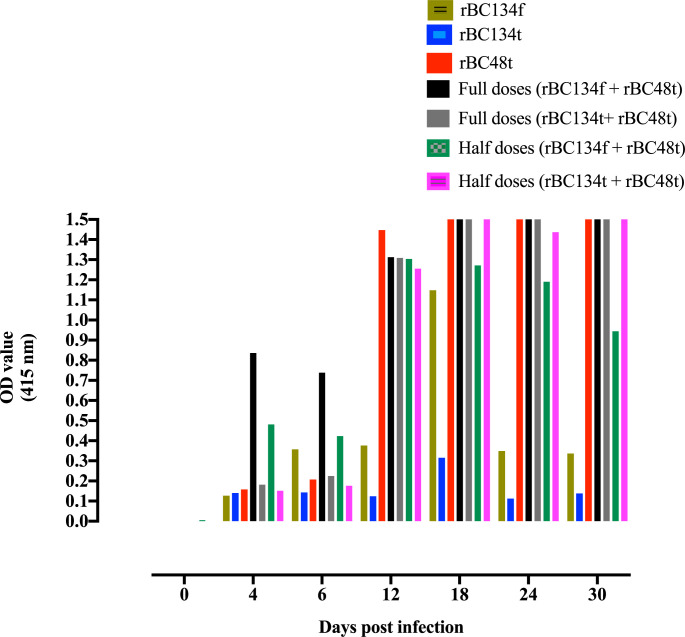
Reactivity of ELISA using recombinant proteins with serially collected sera from a horse experimentally infected with *B*. *caballi*.

Serially collected sera from a horse infected with *B*. *caballi* were used to test the diagnostic abilities of these recombinant proteins. Interestingly, this horse exhibited a high antibody response titer to a cocktail formula full dose (rBC134f+ rBC48t) followed by a half dose of the same cocktail of antigens at Day 4 post infection, and this high antibody titer was maintained until 30 days post infection (Fig 2).

For field serum samples, out of 40 serum samples collected from South Africa, 14, 15, and 20 samples exhibited higher ODs than the cutoff value of rBC134f, rBC134t, and rBC48t, respectively. For the samples collected from Ghana, 19, 16, and 3 exposed higher ODs than the cutoff value of rBC134f, rBC134t, and rBC48t, respectively. Out of 40 samples collected from Mongolia, 5, 5, and 19 showed higher ODs than the cutoff value of rBC134f, rBC134t, and rBC48t, respectively. 13, 13, 13 samples from Thailand were found to be positive for rBC134f, rBC134t, and rBC48t, respectively, while for 40 samples collected from China 12, 7, 21 were found to be positive for rBC134f, rBC134t, and rBC48t, respectively (Table 2). All recombinant proteins used in this study demonstrated good performance as iELISA antigens are capable of identifying the infection. For cocktail antigens of full dose (rBC134f+ rBC48t), full dose (rBC134t+rBC48t), half dose (rBC134f+rBC48t), and half dose (rBC134t +rBC48t) out of 40 samples collected from South Africa 21, 19, 8, 13 samples were found to be positive respectively. While for 40 samples collected from Ghana 24, 11, 18, and 12 were found to be positive. Furthermore, the number of positive samples from Mongolia were found to be 24, 5, 19, and 26 respectively and for Thailand collected samples 15, 14, 11, and 10 were higher than the cutoff value of cocktail antigens and finally out of 40 samples collected from China, 18, 10, 20, 13 samples were found to be positive for different cocktail antigens, respectively (Table 3).

**Table 2:** Summary of iELISA and IFAT results with single recombinant protein results using field sera collected from different locations endemic for *B*. *caballi*.

Site or IFAT result	No. of IFAT samples	No. of iELISA samples					
		rBC134f		rBC134t		rBC48t	
		**+**	**-**	**+**	**-**	**+**	**-**
**South Africa**							
**+**	23	14	9	15	8	20	3
**-**	17	5	12	9	8	9	8
**Total**	40	19	21	24	16	29	11
**Ghana**							
**+**	28	19	9	16	12	3	25
**-**	12	6	6	4	8	0	12
**Total**	40	25	15	20	20	3	37
**Mongolia**							
**+**	29	5	24	5	24	19	10
**-**	11	0	11	0	11	1	10
**Total**	40	5	35	5	35	20	20
**Thailand**							
**+**	20	13	7	13	7	13	7
**-**	20	3	17	3	17	3	17
**Total**	40	16	24	16	24	16	24
**China**							
**+**	22	12	10	7	15	21	1
**-**	18	0	18	9	9	3	15
**Total**	40	12	28	16	24	24	16
**Overall**							
**+**	122	63	59	56	66	76	46
**-**	78	14	64	25	53	16	62
**Total**	200	77	123	81	119	92	108

**Table 3:** Summary of iELISA and IFAT results with cocktail recombinant protein results using field sera collected from different locations endemic for *B*. *caballi*.

Site or IFAT result	No. of IFAT samples	No. of iELISA samples							
		Full doses (rBC 134f + rBC 48t)		Full doses (rBC 134t+ rBC 48t)		Half doses (rBC 134f + rBC 48t)		Half doses (rBC 134t + rBC 48t)	
		**+**	**-**	**+**	**-**	**+**	**-**	**+**	**-**
**South Africa**									
**+**	23	21	2	19	4	8	15	13	10
**-**	17	2	15	3	14	4	13	7	10
**Total**	40	23	17	22	18	12	28	20	20
**Ghana**									
**+**	28	24	4	11	17	18	10	12	16
**-**	12	7	5	3	9	7	5	3	9
**Total**	40	31	9	14	26	25	15	15	25
**Mongolia**									
**+**	29	24	5	5	24	19	10	26	3
**-**	11	1	10	1	11	3	8	3	8
**Total**	40	25	15	6	35	22	18	29	11
**Thailand**									
**+**	20	15	5	14	6	11	9	10	10
**-**	20	3	17	3	17	2	18	3	17
**Total**	40	18	22	17	23	13	27	13	27
**China**									
**+**	22	18	4	10	12	20	2	13	9
**-**	18	2	16	0	18	3	15	0	18
**Total**	40	20	20	10	30	23	17	13	27
**Overall**									
**+**	122	102	20	59	63	76	46	74	48
**-**	78	78	15	63	10	69	19	59	16
**Total**	200	117	83	69	132	95	105	90	110

The specificity and sensitivity of iELISAs with serum samples derived from *B*. *caballi*–endemic areas were determined based on IFAT results as a reference test (Tables 3 & 4). The specificity results were 85.55%, 83.33%, and 83.34%, for rBC134f, rBC134t, and rBC48t, respectively while the specificity results for cocktail antigens were 84.45%, 91.11%, 80%, and 83.33%for full dose (rBC134f+ rBC48t), full dose (rBC134t+rBC48t), half dose (rBC134f+rBC48t) and half dose (rBC134t+rBC48t) (Table 4). For the sensitivity results 40.80%, 27.20%, and 50.40% for rBC134f, rBC134t, and rBC48t, respectively, Additionally, 71.20%, 40.80%, 48%, and 48.10% were the sensitivity results for a cocktail antigen, full dose (rBC134f+ rBC48t), full dose (rBC134t+rBC48t), half dose (rBC134f+ rBC48t) and half dose (rBC134t+ rBC48t) respectively (Table 4). Moreover, a cocktail antigen of full dose (rBC134f+ rBC48t) showed the highest kappa value among the used antigens (Table 4). The agreement (concordance) between the IFAT and the iELISA was higher in cocktail antigen than single antigen and astonishingly the concordance of half dose (rBC134t+ rBC48t) cocktail antigen is higher than the full dose (rBC134t+ rBC48t) and all single antigens (Table 4). These results confirmed the high diagnostic performance and accuracy of the used full dose (rBC134f+ rBC48t) cocktail antigen for the detection of the *B*. *caballi* infection in horses in early stage of infection and in the differentiation between *B*. *caballi* and *T*. *equi* infections.

**Table 4:** The specificity and sensitivity of iELISAs and a comparison of iELISA and IFAT results.

Parameters	rBC134f	rBC134t	rBC48t	Full doses (rBC 134f + rBC 48t)	Full doses (rBC 134t+ rBC 48t)	Half doses (rBC 134f + rBC 48t)	Half doses (rBC 134t + rBC 48t)
**Specificity (%)**	85.55	83.33	83.34	84.45	91.11	80.00	83.33
**Sensitivity (%)**	40.80	27.20	50.40	71.20	40.80	48.00	48.10
**Concordance (%)**	59.53	50.70	64.19	76.74	61.86	61.40	62.26
**Kappa value**	0.51	0.35	0.58	0.79	0.42	0.58	0.55

## Discussion

The ability to diagnose *B*. *caballi* infection in animals is definitely important for proper and accurate treatment and prevention of further hemo-parasites transmission to susceptible animals. Therefore, laboratory diagnosis of equine piroplasmosis is a vital step, due to non-specific clinical signs in acute form and the absence of clinical signs in most of the infected animals. The most used diagnostic assays is microscopy, which is useful for the detection of the acute infections, however, it is less sensitive, and therefore not suitable for detecting asymptomatic carriers [19]. For Indirect immunofluorescence and immunosorbent assays, animals that are persistently infected or with active infection will show high antibody titers, while in animals that eliminate the agent, antibodies will decrease to the point where they cannot be detected in a period of six to ten months [3, 10, 11]. The polymerase chain reaction technique for *B*. *caballi* and *T*. *equi* is highly efficient, however, time and cost of implementation are two inconvenient aspects of this technique [20]. In addition it was reported that the global molecular prevalence of EP, is considerably lower than its seroprevalence especially in *B*. *caballi* (8% versus 20%) [2]. This may be attributed to the parasite clearance, with persistence of antibodies, and may also be due to inherent difficulty of detecting parasite DNA when parasitemia is low [2].

Several studies on ELISA based on RAP1 and EMA1/EMA2 recombinant antigens have been conducted for serodiagnosis of *B*. *caballi* and *T*. *equi*, respectively using competitive ELISAs (cELISAs) [21]. However, recent studies reveals false negative results which might be associated with *B*. *caballi* cELISA due to high genetic diversity of RAP1 and with *T*. *equi* cELISA due to the absence of EMA1 in *Theileria haneyi* [9]. To overcome the genetic diversity might be helped by the presence of multiple epitopes from different antigens in the immunoassay to detect the antibodies during different stages of the infection [8, 10, 11]. Hence, combining two or more recombinant proteins may increase the sensitivity of recombinant iELISAs, as has been previously determined for the diagnosis of different parasites [3, 7, 11]. Currently, recombinant proteins are used to detect the infection rather than using a serological test with a crude antigen prepared from merozoites [7, 18]. A previous study by Ikadai et al., [22] concluded that the 48-kDa protein is immunodominant and might be an important antigen for diagnosis and was reported to be involved in the invasion of erythrocytes [13] and to differentiate very clearly between *B*. *caballi*-infected horse sera, and *T*. *equi*-infected horse sera. Moreover, an ELISA using a newly identified rBC134 protein was developed and proved to be highly specific for the detection of *B*. *caballi*-specific equine antibodies [23]. Therefore, these proteins were considered to be suitable antigens for use in immunodiagnostic tests for *B*. *caballi* infection. Subsequently, in the present study, we selected BC134 and BC48 to truncated their sequencing for developing newly truncated antigens and used them in different cocktail formulas and evaluated the diagnostic performance, validity, and accuracy of these formulas for the detection of *B*. *caballi* -infection in comparison with the single antigen in horses. Interestingly, the obtained findings revealed that the best and highest concordance and kappa values were obtained by cocktail antigen of full dose (rBC134f+rBC48t) between all used antigens and remarkably followed by the single BC48t antigen. Of note, the obtained sensitivity of full dose (rBC134f+rBC48t) cocktail antigen (71.20%) is nearly similar to 73% sensitivity of RAP1 [21], and 79% sensitivity of MAb BC5.37.70.27 (BC5) of IgM type [24]. Importantly, the full dose (rBC134f+rBC48t) cocktail antigen can detect *B*. *caballi* infection in sera from an experimentally infected horse from the 4^th^ day post-infection and was still positive for 1 month after infection. Moreover, it successfully detects infection in field samples collected from different locations endemic for *B*. *caballi*. This highly diagnostic performance of full dose (rBC134f+rBC48t) cocktail antigen can be attributed to the fact that cocktail antigen gathers advantages of both BC134 and BC48 antigens. The first one is that BC134 protein was observed in the cytoplasm and/or membrane of the infected erythrocytes as well as in the cytoplasm at all developmental stages of the intracellular parasites (the ring and subsequent pear-shaped forms) and free merozoites [13]. Moreover, BC134 protein seemed to have interacted with the cytoskeleton and/or the membrane of erythrocytes at the later phase of the parasite development, which denote that it can be detected during different stages of the parasite. Meanwhile, the BC48- proteins were confirmed to be major antigens of *B*. *caballi* merozoites [22]. Therefore, taken together these features of both BC134 and BC48 might explain the best diagnostic performance of this cocktail antigen when used in a full dose. The obtained results were in accordance with those observed in similar studies which used cocktail antigen for the detection of the infection caused by *T*. *equi* in horse and *B*. *bovis* in cattle [3, 7]. Although, the present study highlighted the promising application of rBC134f+ rBC48t cocktail antigen for global detection of *B*. *caballi* infection in horses, future studies are required to elucidate the presence of cross reaction of this antigen with other apicomplexan pathogens such as *Toxoplasma*, *Neospora* etc.

## Conclusion

The obtained results indicated that the cocktail formula of (rBC134f+ rBC48t) antigen is a promising global antigen in the diagnosis of *B*. *caballi* infection in both acute and chronic stages of infection and as early as 4 days post-infection and beyond 1 month after infection with the best and highest concordance and kappa values when compared to other cocktail formula used in this study.

## Supporting information

S1 Fig Successful expression of recombinant proteins in *E*. *coli*. Twelve percent SDS-polyacrylamide gel electrophoresis (SDS-PAGE) of recombinant protein stained with Coomassie blue. Lanes: M, molecular mass marker; 1, rBC134f; 2, rBC134t; 3, rBC48t. The size of each recombinant protein is indicated on the right.
(PDF)

S2 Fig Western blot analysis with horseradish peroxidase-conjugated goat anti-horse IgG against different recombinant proteins. Lane 1, GST (negative control). Lane 2, rBC134f. Lane 3, rBC134t. Lane 4, rBC48t.
(PDF)

S1 Dataset (XLSX)

S1 Raw images (PDF)
