# Supplementary figures and images for: Development of a promising antigenic cocktail for the global detection of Babesia caballi in horse by ELISA
Source: PLoS One. 2023 Apr 14;18(4):e0284535. doi: 10.1371/journal.pone.0284535 (PMC10104287; doi:10.1371/journal.pone.0284535)

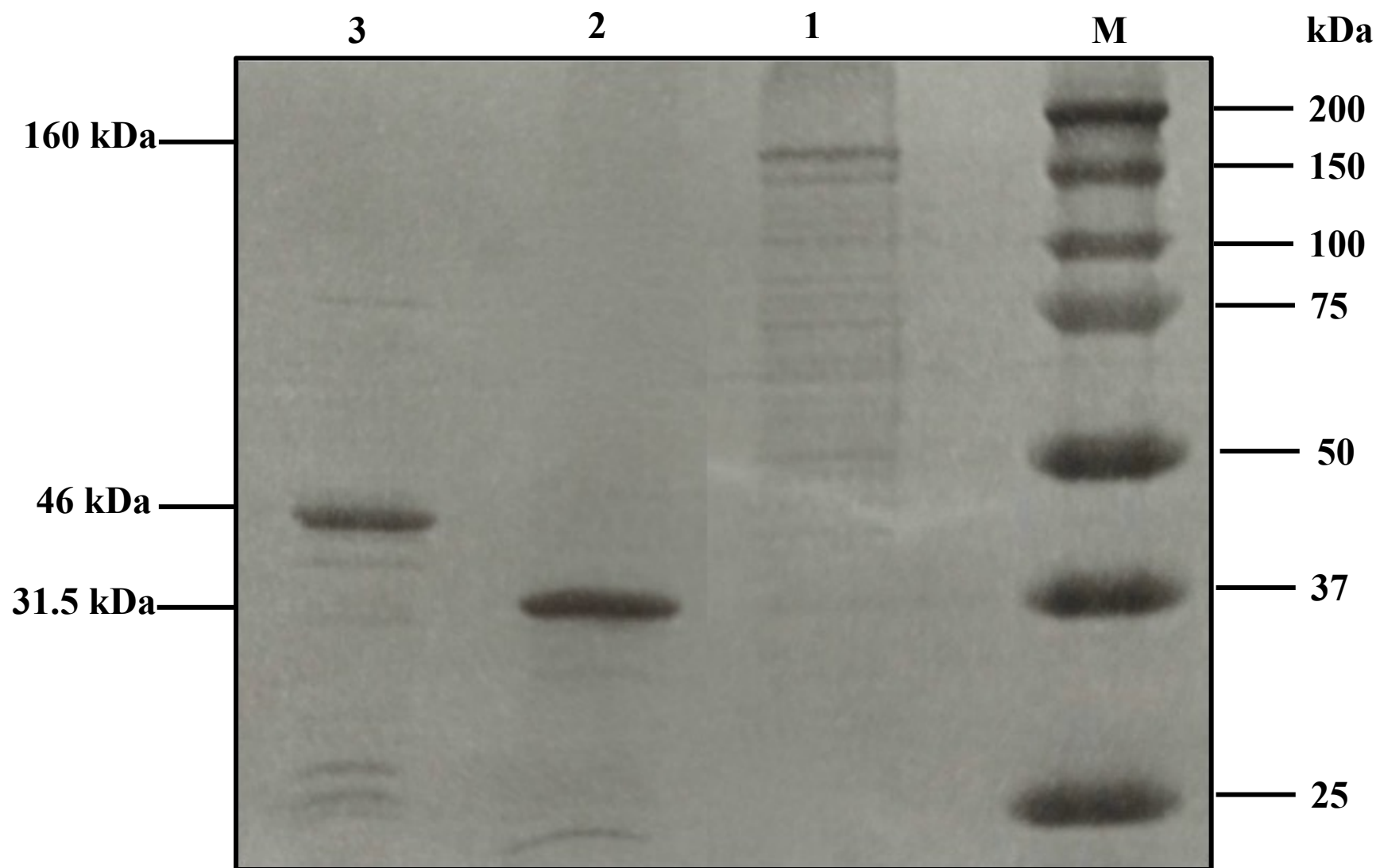

Supplement: S1 Fig — Twelve percent SDS-polyacrylamide gel electrophoresis (SDS-PAGE) of recombinant protein stained with Coomassie blue. Lanes: M, molecular mass marker; 1, rBC134f; 2, rBC134t; 3, rBC48t. The size of each recombinant protein is indicated on the right. (PDF) [file pone.0284535.s001.pdf]

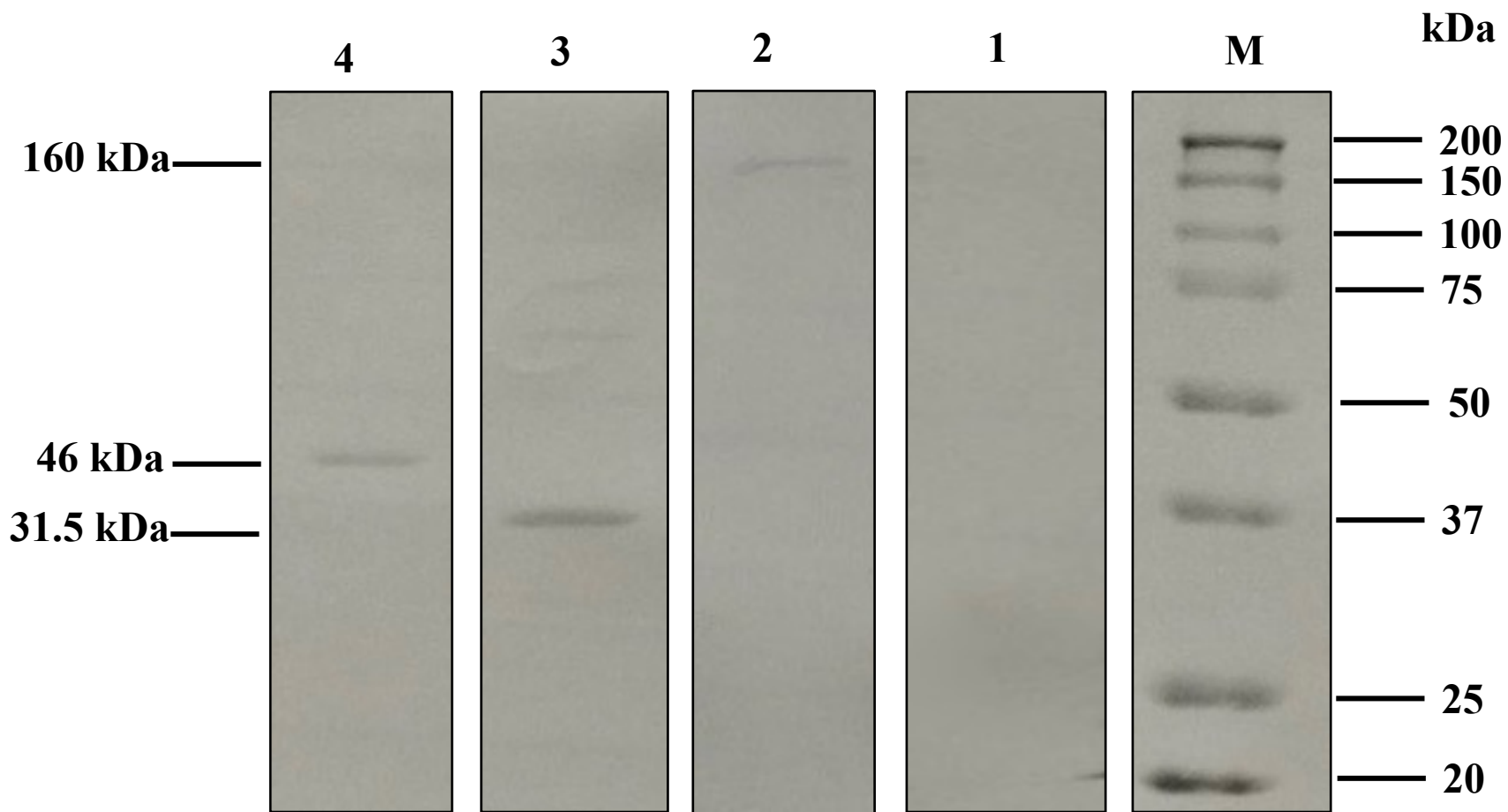

Supplement: S2 Fig — Lane 1, GST (negative control). Lane 2, rBC134f. Lane 3, rBC134t. Lane 4, rBC48t. (PDF) [file pone.0284535.s002.pdf]

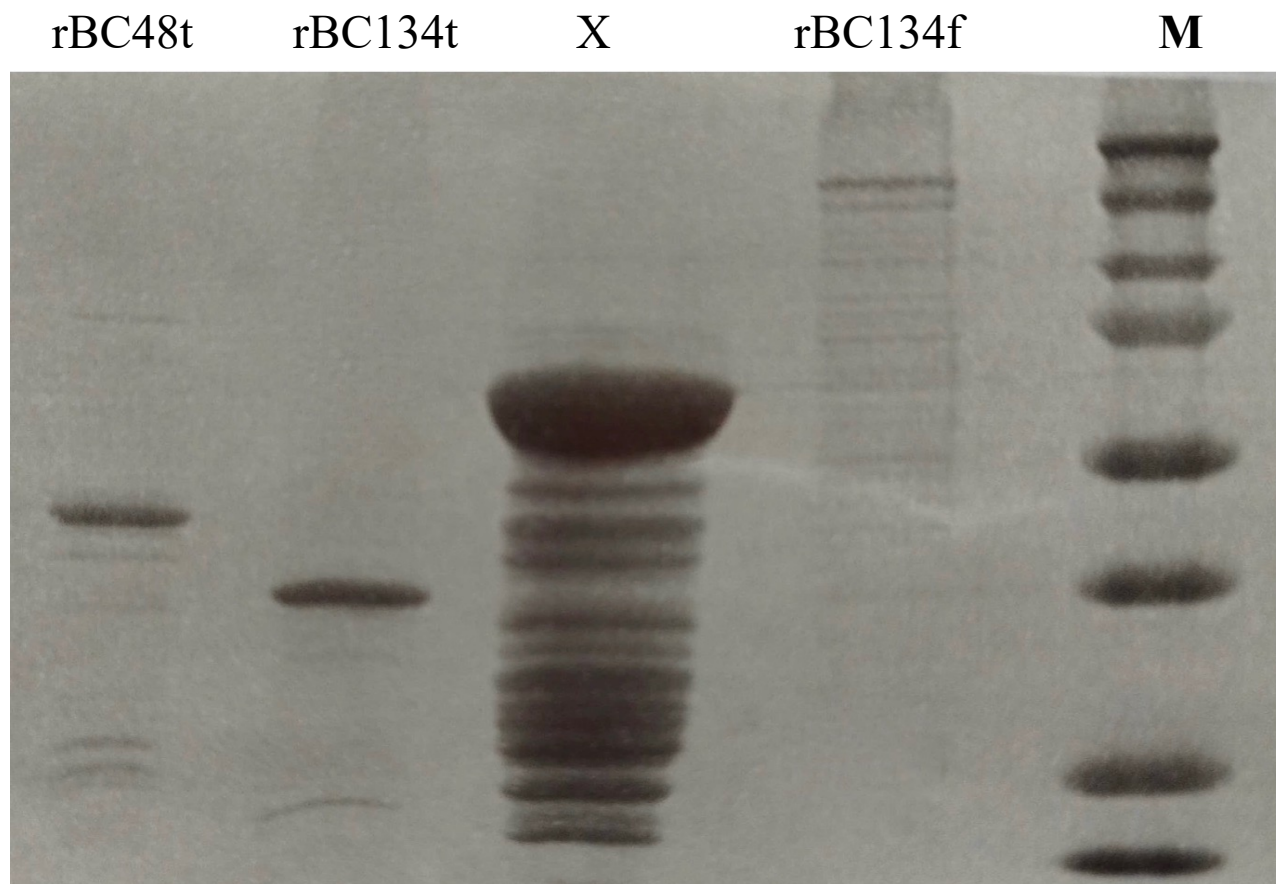

SDS-polyacrylamide gel electrophoresis of recombinant protein

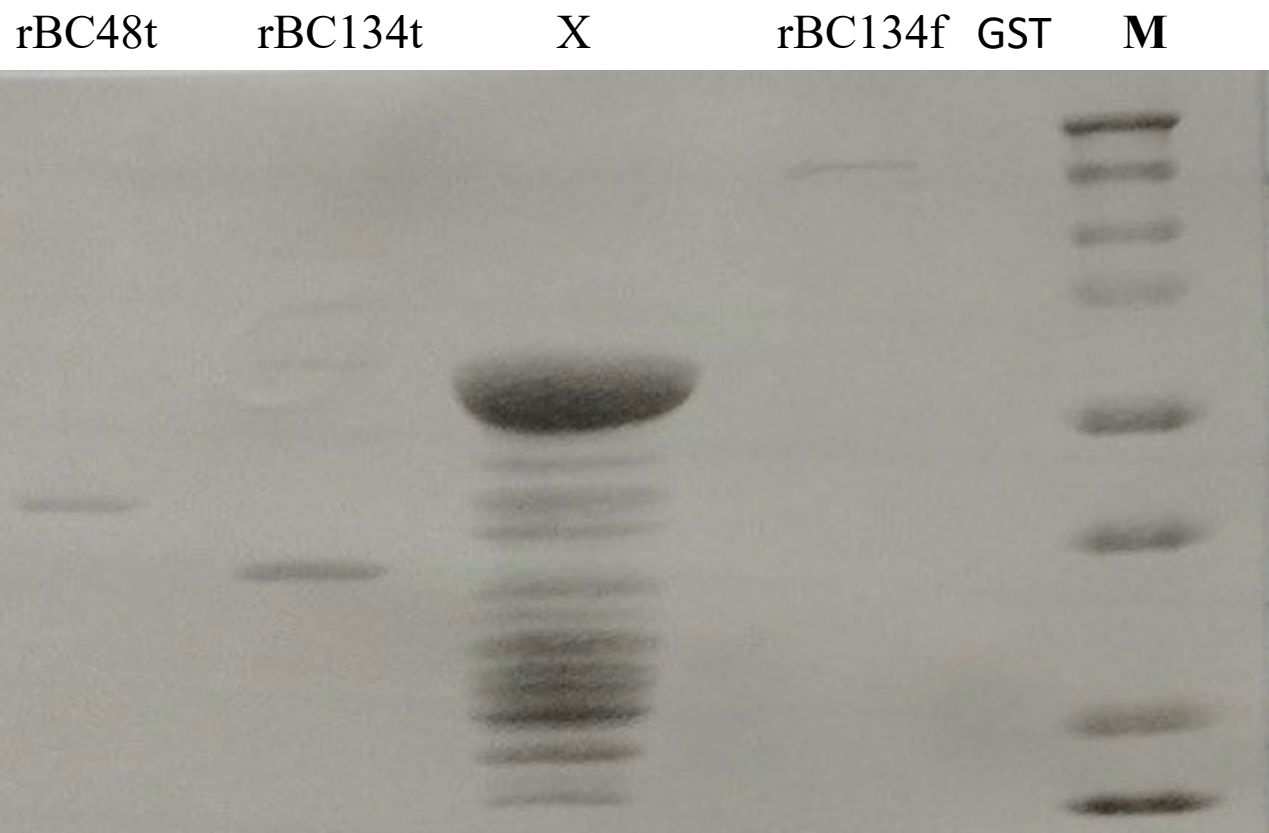

Western blot analysis

Supplement: S1 Raw images — (PDF) [file pone.0284535.s004.pdf]
